# Nanobody-targeted E3-ubiquitin ligase complex degrades nuclear proteins

**DOI:** 10.1038/srep14269

**Published:** 2015-09-16

**Authors:** Yeong Ju Shin, Seung Kyun Park, Yoo Jung Jung, Ye Na Kim, Ki Sung Kim, Ok Kyu Park, Seung-Hae Kwon, Sung Ho Jeon, Le A. Trinh, Scott E. Fraser, Yun Kee, Byung Joon Hwang

**Affiliations:** 1 Department of Molecular Bioscience, College of Biomedical Science, Kangwon National University, Chunchon, 200-701, Republic of Korea; 2 Department of Systems Immunology, College of Biomedical Science, Kangwon National University, Chunchon, 200-701, Republic of Korea; 3 Korea Basic Science Institute Chuncheon Center, Chuncheon, 200-701, Republic of Korea; 4 Department of Life Science, Hallym University, Chuncheon, 200-702, Republic of Korea; 5 Biological Sciences and Biomedical Engineering, University of Southern California, Los Angeles, CA, USA

## Abstract

Targeted protein degradation is a powerful tool in determining the function of specific proteins or protein complexes. We fused nanobodies to SPOP, an adaptor protein of the Cullin-RING E3 ubiquitin ligase complex, resulting in rapid ubiquitination and subsequent proteasome-dependent degradation of specific nuclear proteins in mammalian cells and zebrafish embryos. This approach is easily modifiable, as substrate specificity is conferred by an antibody domain that can be adapted to target virtually any protein.

Our understanding of biomolecular pathways has been rapidly advanced by technologies that modulate protein levels. Manipulations of coding or regulatory gene sequences, and the targeting of mRNA transcripts by RNA interference (RNAi) or Morpholino antisense oligonucleotides, have become frontline tools in determining the function of specific proteins^1^,^2^. These latter techniques decrease protein expression without genomic modification, but are limited by their inefficiency at depleting long-lived proteins, their lack of immediate reversibility, and their potential for off-target effects^3^,^4^.

To overcome these limitations, various approaches have been developed to directly degrade specific proteins. These methods include the addition of different destabilizing domains (degrons) that induce degradation of the tagged proteins following application of drugs or light^5^,^6^. Alternatively, the ubiquitin-dependent proteasome can be utilized to degrade specific protein targets tagged with particular E3 ubiquitin recognition domains^7^,^8^. These approaches all require genetic manipulation of target proteins to introduce tagging domains; however, a recent study demonstrated that engineering E3 ubiquitin ligase itself could control target specificity^9^. In this “deGradFP” technique, GFP is recognized by a modified form of the ‘SKP1-CUL1-F-box’ (SCF) E3 ligase complex in which the substrate recognition domain (WD motifs in F-box protein) is replaced with an anti-GFP nanobody (natural single-domain antibody containing only heavy chains)^10^, resulting in the targeted degradation of proteins containing GFP^9^. In principle, this approach of engineering E3 ubiquitin ligase substrate specificity could be extended to facilitate the targeted degradation of many endogenous proteins, limited only by antibody availability^10^. Here we describe a new method that degrades target nuclear proteins by modifying the substrate specificity of the E3 ubiquitin ligase adapter protein SPOP, and is more efficient than the deGradFP system.

## Results and Discussion

### Development of a nanobody-targeted E3-ubiquitin ligase that specifically degrades nuclear proteins

**Figure 1:**
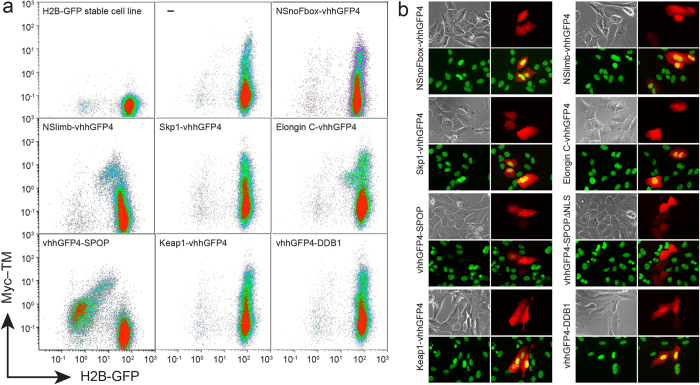
FACS and microscopic analyses of H2B-GFP expression after transient transfection of synthetic E3 ligase candidates. (**a**) FACS analysis of H2B-GFP/293TetOn stable cell line transiently transfected with various synthetic E3 ligase candidates. After transfection, Myc_10_-TM (transmembrane form of Myc_10_ epitope) and each candidate ligase were simultaneously expressed from a bi-directional tetracycline response element (TRE) promoter following doxycycline treatment (Supplementary Fig. 2). vhhGFP4 is a nanobody against GFP. NSnoFbox was derived from NSlimb by deleting its F-box domain necessary for binding to the adaptor protein in the Skp1-Cullin1-F-box E3 ubiquitin ligase complex^9^. ‘–’ represents expression of Myc_10_-TM without any candidate E3 ligase. Only vhhGFP4-SPOP E3 ligase greatly depleted H2B-GFP in the cells expressing Myc_10_-TM. (**b**) Microscopic analysis of H2B-GFP/U2OS stable cell line transiently transfected with the ligase candidates. Doxycycline (1 μg/ml) was administered for 24 hours to promote expression of TagRFP and each ligase candidate. Each panel shows bright field, TagRFP (red), H2B-GFP (green), and merged images of both TagRFP and H2B-GFP. Only vhhGFP4-SPOP or vhhGFP4-SPOPΔNLS has no yellow nuclear signal in merged images because of H2B-GFP depletion in cells expressing TagRFP.

Our approach was motivated by experiments in which the deGradFP method worked poorly in a stable cell line expressing histone H2B (H2B)-GFP (Fig. 1), leading us to design several novel synthetic E3 ligases that could be tested for selective nuclear protein degradation (Supplementary Fig. 1). Cullin-RING E3 ubiquitin ligase (CRL) complexes were selected as the frameworks for designing synthetic ligases, as they are well characterized and directly transfer ubiquitin from the E2 enzyme to the target protein^11^,^12^. The C-terminal region of Cullin binds to RING, while the N-terminal region links to an adaptor protein (Skp1 for Cul1, Elongin B/C for Cul2/5, BTB for Cul3, and DDB1 for Cul4). With the exception of BTB that contains Cullin-binding and substrate recognition domains in the same protein, the adaptor proteins bind to substrate binding proteins, such as F-box proteins for Skp1, VHL/SOCS-box proteins for Elongin B/C, and DCAFs for DDB1. In deGradFP, anti-GFP nanobody (vhhGFP4) was fused to a deletion mutant of NSlimb, a F-box protein, which lacks a substrate-binding domain^9^. To enhance E3 activity in our synthetic ligases, the GFP nanobody was fused directly to a truncated adaptor protein in which domains necessary for interacting with substrate binding proteins, but not the domains for binding to Cullin, were deleted (Supplementary Fig. 1). Thus, the substrate-recognition function of natural E3 ligases was completely replaced with the GFP nanobody in our synthetic ligases.

**Figure 2:**
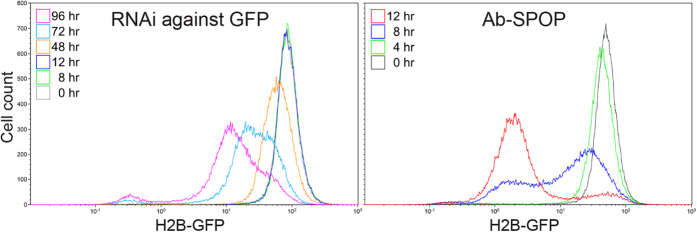
Comparison of RNAi and Ab-SPOP E3 ligase-mediated H2B-GFP depletion. A 293TetOn cell line stably expressing H2B-GFP from a CMV promoter and Ab-SPOP/TagRFP from a bi-directional TRE promoter (Supplementary Figs 5 and 7) was used for the experiment. After GFP siRNA (40 nM) treatment for 0, 8, 12, 48, 72, and 96 hours in the absence of doxycycline, cells were analyzed by FACS for H2B-GFP expression. Doxycycline (1 μg/ml) was added for 0, 4, 8, and 12 hours for FACS analysis of H2B-GFP expression.

To identify the cells expressing candidate ligases in transient transfection experiments, we first constructed a vector containing a bi-directional promoter, permitting tetracycline treatment to direct co-expression of both the candidate ligase and TagRFP; in other experiments, the TagRFP was replaced by an synthetic protein to express Myc epitope on the cell membrane (Myc_10_-TM) (Supplementary Fig. 2). We measured nuclear H2B-GFP fluorescence by flow cytometry 24 hours after transfection into 293TetOn cells expressing H2B-GFP, and found that transfection with the synthetic ligase vhhGFP4-SPOP (Ab-SPOP) greatly decreased the GFP signal (~50 fold) in cells expressing Myc_10_-TM (Figs 1a and 2). Elongin C-vhhGFP4 and NSlimb-vhhGFP4 (the synthetic ligase developed in deGradFP) decreased the nuclear H2B-GFP signal only slightly (3–5 fold); the other ligase candidates had no effect. We confirmed that Ab-SPOP ligase efficiently depleted H2B-GFP in U2OS cells by fluorescence microscopy (Fig. 1b). In this transfection experiment with ligase candidates, only vhhGFP4-SPOP or vhhGFP4-SPOPΔNLS depleted H2B-GFP in cells expressing TagRFP.

**Figure 3:**
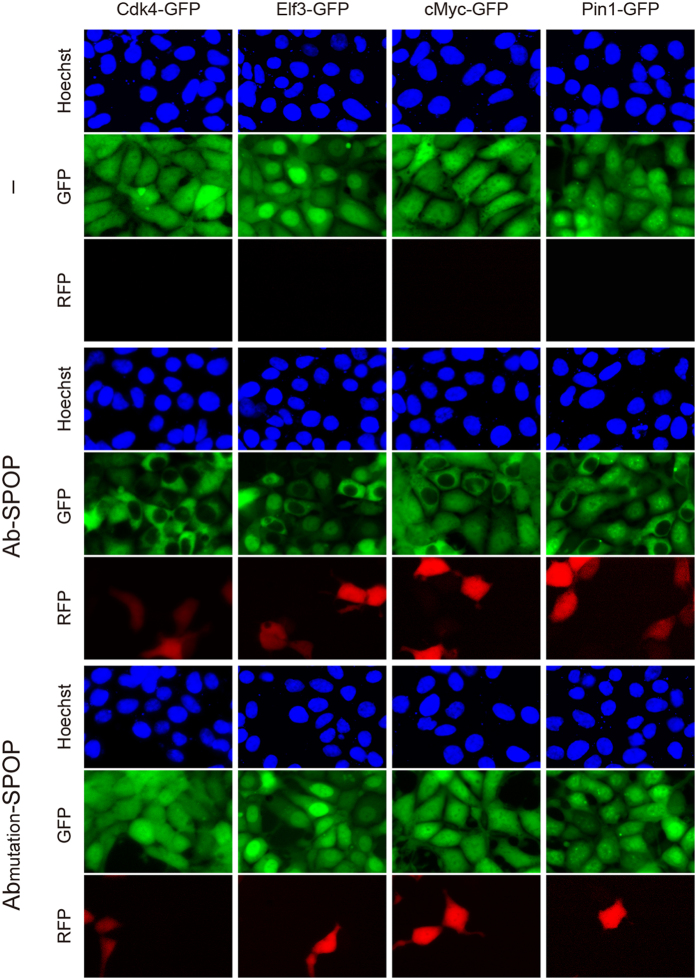
Selective depletion of nuclear GFP-fusion proteins by Ab-SPOP. 293TetOn cells expressing Cdk4-GFP, Elf3-GFP, cMyc-GFP, or Pin1-GFP were transfected with vector expressing Ab-SPOPΔNLS or Ab_mutation_-SPOPΔNLS from a bi-directional TRE promoter. ‘–’ indicates untransfected cells. Expression of TagRFP and depletion of GFP were measured 8 hours after adding doxycycline (1 μg/ml) to media. Selective depletion of GFP in the nucleus, but not cytoplasm, was only observed in cells transfected with Ab-SPOP.

**Figure 4:**
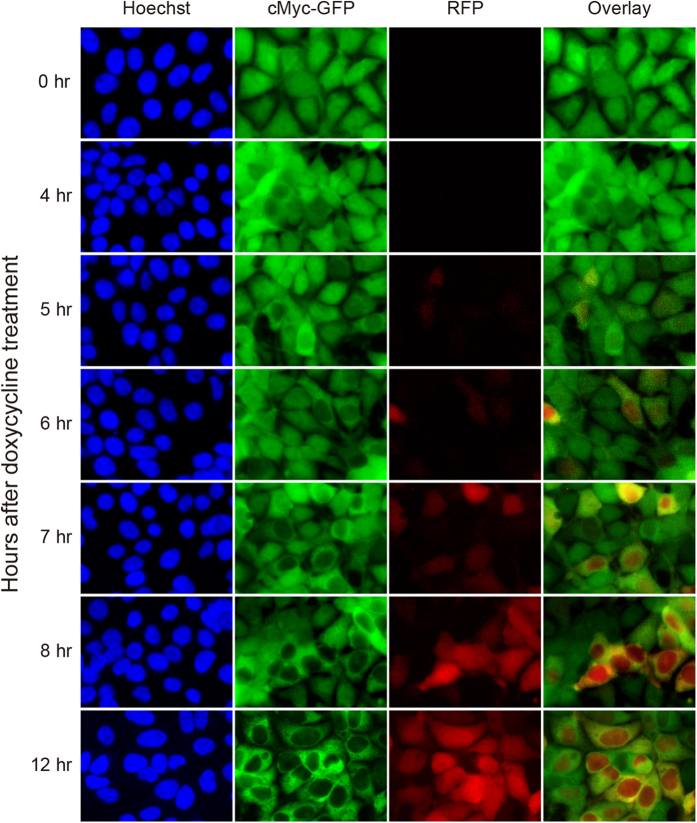
Time course of nuclear cMyc-GFP depletion by Ab-SPOP. 293TetOn cells stably expressing cMyc-GFP from a CMV promoter and RFP/Ab-SPOP from a bi-directional TRE promoter were imaged under epifluorescence at different time points (0, 4, 5, 6, 7, 8, 12 h) after doxycycline (1 μg/ml) administration. Overlay merges images of c-Myc-GFP and RFP.

Ab-SPOP ligase, but not Ab_mutation_-SPOP (in which the GFP-recognition domain of the vhhGFP4 nanobody was deleted), degraded all six GFP fusion proteins that we tested: cMyc-GFP (78 kDa), Pin1-GFP (46 kDa), Cdk4-GFP (61 kDa), Elf3-GFP (69 kDa), and GFP (27 kDa). Interestingly, GFP was only depleted in the cell nucleus; non-nuclear GFP was not affected (Fig. 3 and Supplementary Fig. 3). In all cases, Ab-SPOP ligase promoted depletion of GFP or GFP fusion proteins within 3 hours of doxycycline-induced expression, before TagRFP was detectable under epifluorescence (Fig. 3 and Supplementary Figs 3–6). This selective depletion of GFP fusion proteins before the appearance of the RFP signal was investigated further using a stable cell line containing a CMV promoter driving cMyc-GFP expression, and a bi-directional TRE promoter controlling expression of both TagRFP and Ab-SPOP. In these cells, cMyc-GFP began to be depleted from the nucleus 4–5 hours after doxycycline treatment; TagRFP expression was not detectable at this time point under epifluorescence (Fig. 4). All cells expressed TagRFP and had depleted levels of nuclear cMyc-GFP 12 hours after doxycycline treatment (Fig. 4). Four alternative GFP nanobodies (GBP1, 2, 6 and 7; known to recognize different epitopes in GFP^13^) were also efficient at depleting H2B-GFP and GFP from the nucleus when used in Ab-SPOP ligases in place of vhhGFP4 (Supplementary Figs 4 and 5).Thus Ab-SPOP can degrade a broad spectrum of differently sized nuclear GFP-fusion proteins and the nanobody domain can be altered, indicating this method will be easily adaptable to other targets.

**Figure 5:**
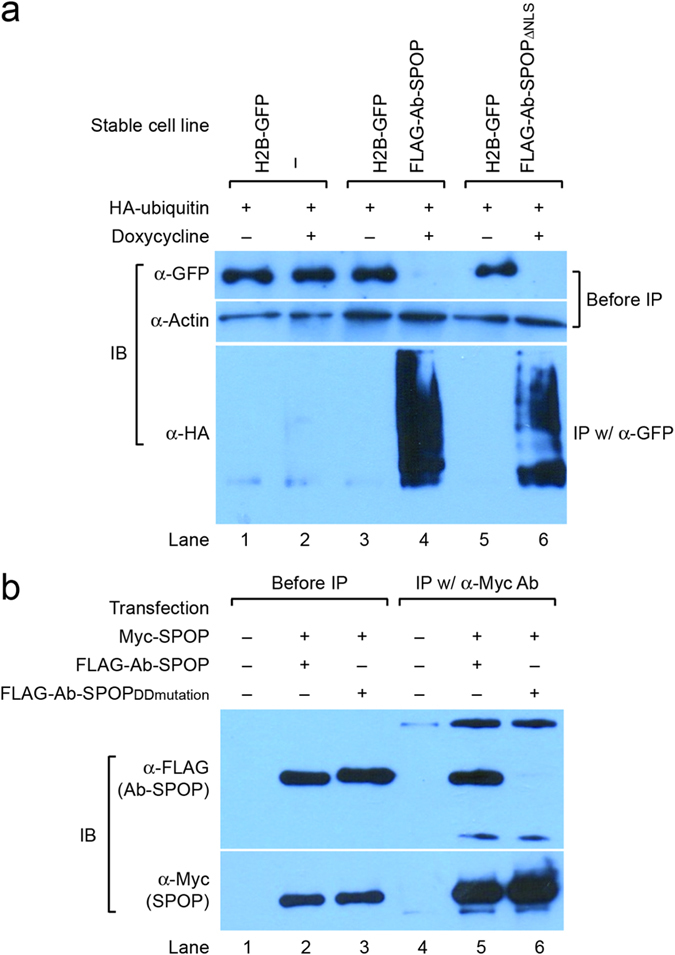
Ubiquitination of H2B-GFP by SPOP/Ab-SPOP dimer. **(a)** 293TetOn cells expressing: (i) H2B-GFP from CMV promoter (ii) H2B-GFP from CMV promoter and FLAG-Ab-SPOP from TRE promoter (iii) H2B-GFP from CMV promoter and FLAG-Ab-SPOPΔNLS from TRE promoter. Cells were transiently transfected with HA-ubiquitin plasmid, the TRE promoter was then activated by doxycycline treatment. Crude cell extracts were prepared and analyzed by western blotting with anti-GFP and anti-actin antibodies to measure the degradation of H2B-GFP by Ab-SPOPs. The same extracts were used for an *in vivo* H2B-GFP ubiquitination assay using immunoprecipitation with anti-GFP antibody and then western blotting with anti-HA antibody. **(b)** Coimmunoprecipitation of SPOP with Ab-SPOP. SPOP tagged with Myc epitope (Myc-SPOP) was co-transfected with FLAG-Ab-SPOP or FLAG-Ab-SPOP_DD mutation_ in HEK 293T cells. The DDmutation prevents SPOP from forming homodimers^14^. After immunoprecipitation with anti-Myc antibody, western blotting was carried out with anti-Myc and anti-FLAG antibodies. Blot shows that Ab-SPOP interacts with SPOP through the DD domain.

**Figure 6:**
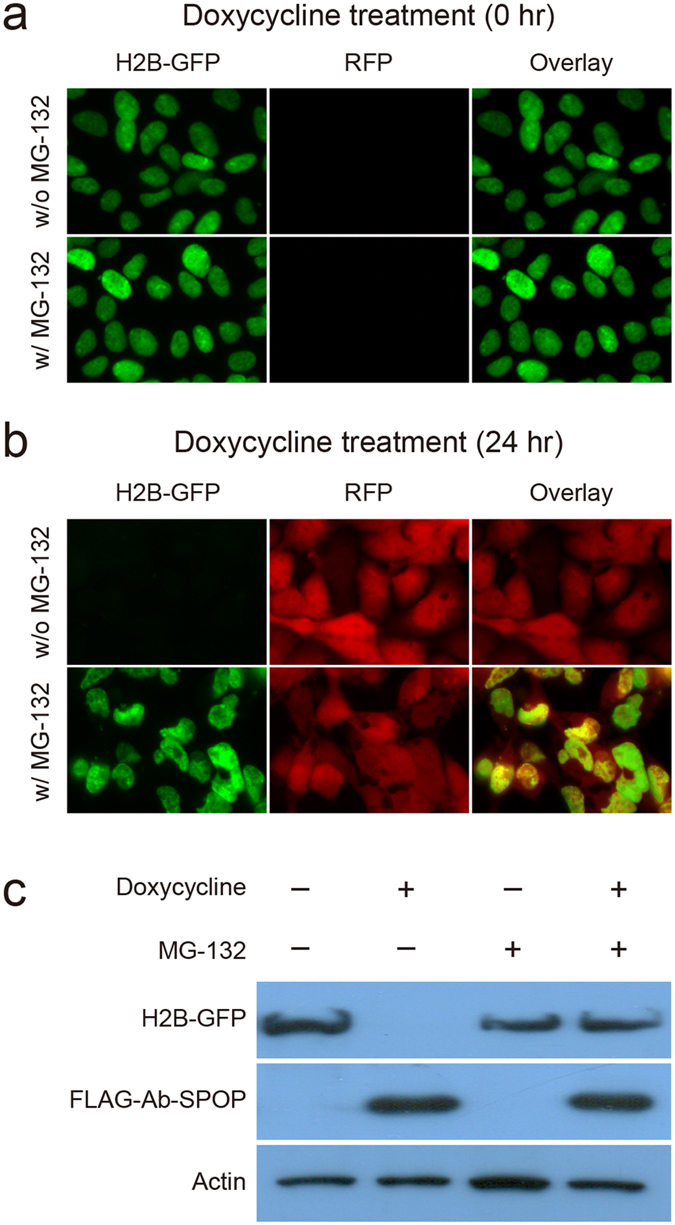
The proteasome inhibitor MG-132 blocks H2B-GFP degradation by Ab-SPOP. 293TetOn cells expressing H2B-GFP from a CMV promoter and FLAG-Ab-SPOP/RFP from a bi-directional TRE promoter were examined under epifluorescence 0 h **(a)** or 24 h **(b)** after doxycycline (1 μg/ml) and MG-132 (5 μg/ml) treatments. **(C)** H2B-GFP and FLAG-Ab-SPOP proteins levels were also investigated by immunoblotting with anti-GFP, anti-FLAG, and anti-actin antibodies.

**Figure 7:**
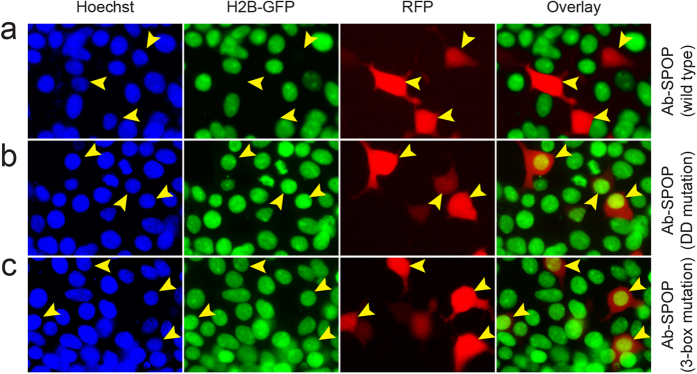
Ab-SPOP_DDmutation_ and Ab-SPOP_3-box mutation_ do not deplete nuclear H2B-GFP. 293TetOn cells expressing H2B-GFP were transfected with vector expressing Ab-SPOP, Ab-SPOP_DDmutation_, or Ab-SPOP_3-box mutation_ from a bi-directional TRE promoter. **(a)** Ab-SPOP causes depletion of nuclear H2B-GFP in cells expressing TagRFP (yellow arrowheads). **(b)** Ab-SPOP_DDmutation_ does not cause depletion of nuclear H2B-GFP in cells expressing TagRFP (yellow arrowheads). This suggests that the Ab-SPOP dimerization domain is necessary for the depletion of H2B-GFP. **(c)** Ab-SPOP_3-box mutation_ does not cause depletion of nuclear H2B-GFP in cells expressing TagRFP (yellow arrowheads). This suggests that the Ab-SPOP 3-box domain is necessary for nuclear depletion of H2B-GFP. The 3-box domain is required for binding with Cul3 in the CRL3 E3 ligase complex^14^, indicating that Ab-SPOP interacts with Cul3 protein for its E3 ubiquitin ligase activity.

To confirm that Ab-SPOP functions as an E3 ubiquitin ligase, we performed an *in vivo* ubiquitination assay. Co-immunoprecipitation showed that Ab-SPOP transfection results in the attachment of a polyubiquitin chain to H2B-GFP, causing its degradation (Fig. 5a). H2B-GFP degradation was blocked by MG-132, a proteasome inhibitor that did not affect the expression of TagRFP and FLAG-Ab-SPOP (Fig. 6). Furthermore, deletion of the 3-box motif (Ab-SPOP_3-box mutation_) responsible for SPOP interaction with Cullin^14^ abolished Ab-SPOP activity (Fig. 7). Interestingly, removal of the nuclear localization signal (NLS) from the C-terminal of Ab-SPOP did not prevent ubiquitylation of H2B-GFP and depletion of nuclear H2B-GFP (Figs 1b and 5a), suggesting that Ab-SPOP forms a heterodimer with endogenous SPOP to generate an active E3 ligase complex that targets nuclear proteins. This hypothesis is supported by the fact that SPOP is not functional as monomer^14^, and is targeted to the nucleus through the NLS at its C-terminal end^15^,^16^. Additionally, mutation of Ab-SPOP’s dimerization domain (Ab-SPOP_DDmutation_) prevents interaction with SPOP and H2B-GFP degradation (Figs 5b and 7), and Ab-SPOP lacking a NLS still depletes target proteins in the cell nucleus, but not in cytoplasm (Figs 3, 4 and Supplementary Figs 3–4). Since protein localization is a dynamic process, nuclear depletion of ubiquitously expressed proteins by Ab-SPOP could also indirectly affect protein concentrations in the cytoplasm, although our current epifluorescent assay is not sensitive enough to measure these changes.

### Comparison of RNAi and Ab-SPOP E3 ligase mediated H2B-GFP depletion

Ab-SPOP-mediated targeted protein depletion was more efficient than a standard RNAi approach (Fig. 2). We performed test experiments in a stable cell line that expresses H2B-GFP from a CMV promoter; in addition, this line expressed Ab-SPOP/TagRFP under the control of a tetracycline-inducible, bi-directional promoter. H2B-GFP intensity was measured by flow cytometry after treatment with siRNA against GFP, or after inducing Ab-SPOP expression with doxycycline. We chose a siRNA sequence that has been widely used to inhibit GFP expression^17^. As expected, RNAi treatment took longer than 72 hours to knockdown the H2B-GFP expression 5–10 fold. In contrast, Ab-SPOP induction decreased GFP signal more than 50-fold within 12 hours of doxycycline application (Fig. 2). When Ab-SPOP was highly expressed, using transient transfection, H2B-GFP levels were visibly depleted 3 hours after induction (Supplementary Fig. 6). Following withdrawal of tetracycline from media, H2B-GFP protein and fluorescent signal only partially recovered after 48 hours, suggesting that Ab-SPOP remains functional as an E3 ligase for approximately 2 days (Supplementary Fig. 7).

### Targeted depletion of Hmga2-Citrine in zebrafish embryos

**Figure 8:**
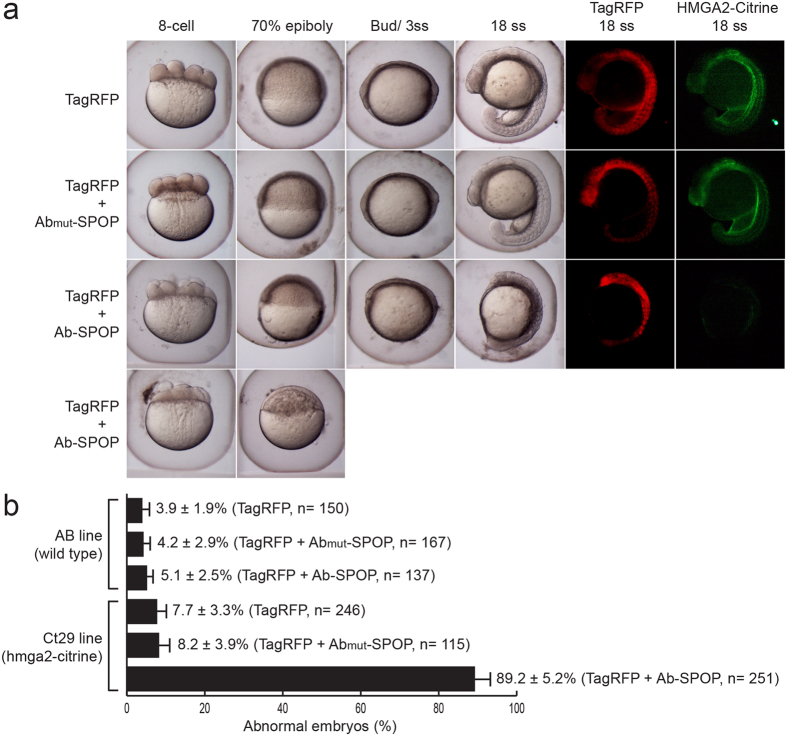
Targeted depletion of Hmga2-Citrine in zebrafish embryos. (**a**) mRNA of wild type Ab-SPOP or mutant Ab(mut)-SPOP (deletion of CDR3 region of the vhhGFP4 nanobody), was injected into one-cell stage zebrafish embryos expressing Hmga2-Citrine protein (homozygous *ct29a*^*GT*^). TagRFP mRNA was co-administered as an injection control. Ab-SPOP expression abolishes Hmga2-Citrine, resulting in early developmental defects such as abnormal cell division during cleavage, delayed cell migration during epiboly and ultimately embryonic death. In contrast, embryos injected with Ab(mut)-SPOP mRNA did not affect Hmga2-Citrine level or disrupt embryonic development, suggesting that Ab-SPOP activity directly promotes degradation of Hmga2-Citrine protein resulting in developmental phenotypes. **(b)** The toxicity of Ab-SPOP was tested by injecting Ab-SPOP mRNA into wild type embryos (AB line). Embryonic development was normal. ‘n’ represents the total number of embryos from several injection experiments.

Since Ab-SPOP rapidly depletes target proteins *in vitro*, we hypothesized it could deplete target proteins during early embryogenesis, even maternally inherited proteins. We used the zebrafish gene trap line *Gt(Hmga2-Citrine)*^*ct29a*^ to test the effectiveness of Ab-SPOP *in vivo*: Hmga2 protein modulates nucleosome and chromatin structure, making it a good candidate for functional protein degradation experiments. Microinjection of Ab-SPOP mRNA (n = 251 embryos, 7 independent experiments), but not Ab_mutation_-SPOP mRNA (n = 361 embryos, 10 independent experiments), into homozygous *ct29a*^*GT*^embryos at the 1-cell stage (injection: 100 pg Ab-SPOP RNA; 10 pg TagRFP RNA) depleted Hmga2-Citrine and caused various defects during early embryogenesis (Fig. 8). Control experiments, microinjecting Ab-SPOP (n = 137 embryos, 3 independent experiments) or Ab_mutation_-SPOP mRNA (n = 317 embryos, 6 independent experiments) into wild type AB embryos (injection: 100 pg Ab-SPOP RNA; 10 pg TagRFP RNA per 1-cell stage embryo), did not cause defects (Fig. 8). These experiments indicate that Ab-SPOP is not toxic during zebrafish development and rapidly depletes GFP fusion proteins *in vivo*.

Ab-SPOP ligase could potentially be used to selectively deplete proteins in the nuclei of all knock-in organisms that contain in-frame fusion proteins with GFP or GFP derivatives. More than 170 FlipTrap zebrafish lines expressing in-frame Citrine fusion proteins have been generated, the majority of which (94.2%) do not exhibit any phenotype and are homozygously viable^18^. Ab-SPOP mediated protein depletion will be a valuable tool in determining the function of the Citrine fusion proteins in these and other knock-in zebrafish lines. These methods will synergize with genome editing techniques, particularly those using CRISPR (Clustered Regularly Interspaced Short Palindromic Repeats)/Cas-mediated site-specific genome nuclease^19^,^20^. Recent findings that morpholino-modified oligonucleotides, widely used in zebrafish research, cause significant off-target effects, highlights the importance of developing alternative methodologies for investigating gene function^3^.

### Concluding remarks

We replaced the substrate recognition domain of SPOP with a nanobody to create Ab-SPOP, a synthetic E3 ubiquitin ligase that polyubiquitinates target nuclear proteins leading to proteasomal degradation. Ab-SPOP depletes H2B-GFP more rapidly and effectively than RNAi, suggesting that targeted protein degradation may provide advantages over traditional gene knock-down methods. We name this approach of using synthetic E3 ligase or protease as ‘Protein interference’ (Protein-i), distinguishing it from degron and RNAi. Further modifications of Ab-SPOP and the manipulation of related E3 ligases will lead to the development of new reagents targeting diverse proteins within different intracellular compartments.

## Methods

### Cell culture

HEK293T, 293TetOn, and U2OSTetOn cells were purchased from ATCC and Clontech. The cells were cultured in DMEM supplemented with 10% FBS and 1% penicillin/streptomycin. They were transiently transfected with Polyethylenimine (PEI) or with JetPRIME PolyPlus (PolyPlus-transfection.com) according to the manufacturer’s directions. Cells cultured with 2 ml media in 6-well cell culture plates were treated with 2 μg doxycycline, a stable tetracycline analogue. When treating with doxycycline for longer than 24 hours, fresh medium containing doxycycline (1 μg/ml) was added every 24 hours.

To generate stable cell lines expressing GFP or GFP fusion proteins, cells were transfected using PolyPlus and selected in a medium containing 2 μg/ml puromycin. When necessary, stable cells expressing GFP were enriched by fluorescence-activated cell sorting. Stable cell lines expressing Ab-SPOP were selected in medium containing 100 μg/ml hygromycin B.

### Plasmids

The following vectors were obtained from Addgene: pbabe-cyclinD1+CDK4R24C (11129), pRK5-HA-Ubiquitin-WT (17608), GST-Pin1 (19027), pcDNA3-FLAG-DDB1 (19918), T7-Elongin C-pcDNA (19998), TetO-FUW-cMyc (20324), pDisplay-AP-CFP-TM (20861), pIRES-puro-ELF3 (25728), FLAG-Keap1 (28023), pACAGW-H2B-PAGFP-AAV (33000), pcDNA3_NSlimb-vhhGFP4 (35579), pcDNA3_NSnoFbox-vhhGFP4 (35580), pCAG-GBP1-10gly-Ga4DBD (49438), pCAG-Gal4DBD-GBP2 (49439), pCAG-p65AD-GBP6 (49440), pCAG-GBP7-p65AD (49441). Plasmids containing Skp1 (MHS1011-7509413) and SPOP (MHS1010-57540) cDNAs were purchased from Open Biosystems.

### Plasmid construction

Vectors that express 10 copies of Myc epitope on the cell membrane (Myc_10_-TM) were prepared by sequential ligation of PCR products and re-PCR amplification of the ligation mixtures. In brief, PCR fragments of signal peptide (SP), Myc_10_ epitope, and transmembrane domain (TM) were separately prepared. After combining the three PCR fragments by ligation and re-PCR amplification, the final PCR fragment was cloned under a tetracycline-inducible, bi-directional promoter in plasmids that co-express an synthetic E3 ubiquitin ligase (Supplementary Fig. 2).

The following PCR products of candidate synthetic E3 ubiquitin ligases were cloned into vectors that co-express TagRFP or an synthetic SP-Myc10-TM protein from a tetracycline-inducible, bi-directional promoter: vhhGFP4-DDB1_354–1140_, Elongin C_1–87_-vhhGFP4, Keap1_1–134_-vhhGFP4, Skp1_1–127_-vhhGFP4, NSlimb-vhhGFP4, NSnoFbox-vhhGFP4, vhhGFP4-SPOP_167–374_, GBP1-SPOP_167–374_, GBP2-SPOP_167–374_, GBP6-SPOP_167–374_, GBP7-SPOP_167–374_. The Ab-SPOP lacking NLS (vhhGFP4-SPOP_167–368_) had 6 amino acids deleted from its C-terminal end. Deletion of vhhGFP4 from Ab-SPOP and deletion of the CDR 3 region from vhhGFP4 resulted in the creation of Ab_mutation_-SPOP vectors. Ab-SPOP_DDmutation_ and Ab-SPOP_3-box mutation_ constructs were prepared by introducing the corresponding mutations into SPOP through PCR-based mutagenesis^14^.

PCR products of GFP and GFP fusion proteins (H2B, Cdk4, Pin1, Elf3, and cMyc) were cloned under the CMV promoter in the pPuro-CMV vector. Sequences of all constructs were verified by mapping with restriction enzymes and DNA sequencing.

### Microscopy

Images were obtained using an Olympus IX71 inverted microscope equipped with pE-2 LED fluorescence light source, Leica DM IL LED inverted microscope equipped with HBO 100W/2 mercury fluorescence lamp, Olympus SZX16 stereoscope with U-LH100HGAPO 103W/2 mercury fluorescence lamp, or Zeiss upright Imager.A2 with HBO100 103W/2 mercury fluorescence lamp. For the IX71 and DM IL LED, photographs were taken with Andor Clara CCD camera and MetaMorph software. An AxioCam HRc camera and ZEN software were used with the Imager A2 microscope. A TUCSEN TCH-5.0ICE CCD camera and ISCapture software were used for the SZX16 stereoscope. GFP and RFP fluorescence were observed using Chroma High Q filters.

To visualize the expression of Myc_10_-TM on cell membrane, α-Myc antibodies (9E10) and Alexa Fluor 568-conjugated α-mouse IgG antibodies were used, following the same staining protocol described in the section below.

### Flow cytometric fluorescence analysis

To quantitate the expression of Myc_10_-TM on cell membrane, cells were washed with washing buffer (1x PBS with 0.5% BSA and 2 mM EDTA), incubated with α-Myc antibodies (9E10, 2 mg/ml, x500 dilution) in 100 μl of washing buffer at 4 ^o^C for 30 min. After removing the unbound primary antibodies by washing twice with the washing buffer, Cy5-conjugated α-mouse IgG secondary antibodies (Molecular probe, 2 mg/ml, x500 dilution) were added in 100 μl. After incubating at 4 ^o^C for 30 min, the cells were washed twice with 1x PBS.

A Moflo XDP flow cytometer (Beckman Coulter) was used for the analysis. The instrument settings were as follows: log forward scatter (FSC) and log side scatter (SSC) at 440 V. log FL1 fluorescence at 400 V, and log FL10 fluorescence at 450 V. The Cy5 was excited at 633 nm and GFP at 488 nm. The Cy5 fluorescence was collected through a 670/30-nm bandpass filter on the FL10 channel, and the GFP fluorescence was collected through a 529/28-nm bandpass filter on the FL1 channel. The singlet cell population was gated on FSC and SSC. The typical sampling rate was 500 cells per second, and the typical sample size was 50,000 cells per measurement unless otherwise stated. The data were analyzed with Kaluza software (Beckman Coulter).

### Antibodies

Monoclonal antibodies against Myc and HA tags and their HRP-conjugated 2^o^ antibodies were purchased from Roche Applied Science. Monoclonal antibody against FLAG (M2) was purchased from Sigma-Aldrich. Anti-actin and anti-GFP antibodies were purchased from Santa Cruz Biotechnology and Invitrogen/Molecular probe, respectively. For immunoblotting, cells were lysed in lysis buffer. After centrifugation, supernatants were subject to SDS-PAGE, and transferred to polyvinylidene difluoride membranes. Protein bands were detected by enhanced chemiluminescence (ECL).

### Co-immunoprecipitation

HEK293T cells were transfected with plasmids that expressed FLAG-tagged Ab-SPOP and Myc-tagged SPOP. Twenty-four hours after transfection, cells were lysed with lysis buffer, immunoprecipitated with anti-Myc antibodies, and subjected to immunoblotting with anti-Myc and anti-FLAG antibodies.

### *In vivo* ubiquitination assay

H2B-GFP ubiquitination *in vivo* was detected by transfecting HA-ubiquitin plasmid into 293TetOn stable cell line that expresses H2B-GFP from CMV promoter as well as FLAG-Ab-SPOP and TagRFP from a tetracycline-inducible, bi-directional promoter. After the bi-directional promoter was induced for 24 hours by doxycycline treatment, cells were lysed and immunoprecipitated with anti-GFP antibodies. Ubiquitination of H2B-GFP was analyzed by immunoblotting with anti-HA antibodies.

### Small interfering RNA (siRNA)

GFP-specific siRNA (5′-GCAAGCUGACCCUGAAGUUC-3′), which was previously used in various experiments, was purchased from Dharmacon. 293TetOn stable cells expressing H2B-GFP were transfected with siRNA (40 nM) with the use of JetPRIME PolyPlus according to the manufacturer’s directions.

### Zebrafish husbandry

Zebrafish (*Danio rerio*) adults of the wild type AB strain and the transgenic FlipTrap line, *Gt(hmga2-citrine)*^*ct29a*^ were maintained in balanced salt water at 27.5 °C in a 14/10 hour light/dark cycle. Embryos were raised in the incubator at 28.5 °C and staged following standard methods. To establish the homozygous *ct29a*^*GT*^ lines, three heterozygous male fish were mated with five heterozygous females, which weakly express Citrine. After mating, 10 F1 embryos (3 males and 7 females) that strongly express Citrine were grown to adult. Then each adult was backcrossed with AB wild types of the opposite sex and Citrine expression was measured for more than 40 embryos from each mating. Adults were determined to be *ct29a*^*GT*^ homozygous when outcrossing to wild type yielded 100% Citrine positive embryos.

### Mounting zebrafish embryos and imaging

Zebrafish embryos were selected for imaging and positioned in holes made in the 1% agarose bed (Biosesang 9012-36-6) in E3 media (5 mM NaCl, 0.17 mM KCl, 0.33 mM CaCl2, and 0.33 mM MgSO4). For live imaging, bright field and fluorescence images of the AB and transgenic embryos were acquired using an Olympus stereomicroscope SZX9 equipped with Tucsen Sony 5.0 MP Cooled CCD camera.

### mRNA microinjection

To generate full-length capped sense mRNA, plasmid DNA of pCS2-TagRFP, pCS2-Ab-SPOP, or pCS2-Abmutation-SPOP was linearized with Not I, and RNA was synthesized by *in vitro* transcription using a SP6 mMESSAGE mMACHINE kit (Ambion). 100 pg of SPOP mRNA was co-injected with 10 pg of TagRFP mRNA into each embryo of AB wild type or *ct29a*^*GT*^ homozygous embryos at the one-cell stage.

### Ethics statement

The zebrafish experiment was carried out in accordance with the recommendations in the Guide for the Care and Use of the University of Southern California. The protocol was approved by the Institutional Animal Care and Use Committee (IACUC) of the University of Southern California.

## Additional Information

**How to cite this article**: Shin, Y.J. *et al.* Nanobody-targeted E3-ubiquitin ligase complex degrades nuclear proteins. *Sci. Rep.* **5**, 14269; doi: 10.1038/srep14269 (2015).

## Supplementary Material

Supplementary Information
