# Supplementary material for: Nanobody-targeted E3-ubiquitin ligase complex degrades nuclear proteins
Source: Sci Rep. 2015 Sep 16;5:14269. doi: 10.1038/srep14269 (PMC4571616; doi:10.1038/srep14269)
Supplement: Supplementary Information [file srep14269-s1.pdf]

SUPPLEMENTARY INFORMATION

Nanobody-targeted E3-ubiquitin ligase complex degrades nuclear proteins

Yeong Ju Shin[1,7], Seung Kyun Park[1,7], Yoo Jung Jung[2,7], Ye Na Kim[1], Ki Sung Kim[1], Ok Kyu Park[4], Seung-Hae Kwon[4], Sung Ho Jeon[5], Le A. Trinh[6], Scott E. Fraser[6], Yun Kee[2,3,*], and Byung Joon Hwang[1,3,*]

[1]Department of Molecular Bioscience, [2]Department of Systems Immunology, College of Biomedical Science, [3]Institute of Bioscience & Biotechnology, Kangwon National University, [4]Korea Basic Science Institute Chuncheon Center, Chuncheon, 200-701, Republic of Korea, [5]Department of Life Science, Hallym University, Chuncheon, 200-702, Republic of Korea, [6]Biological Sciences and Biomedical Engineering, University of Southern California, Los Angeles, CA, USA

[7]These authors contributed equally to this work.

*Correspondence: bjhwang@kangwon.ac.kr (B.J.H.), yunkee@kangwon.ac.kr (Y.K.)

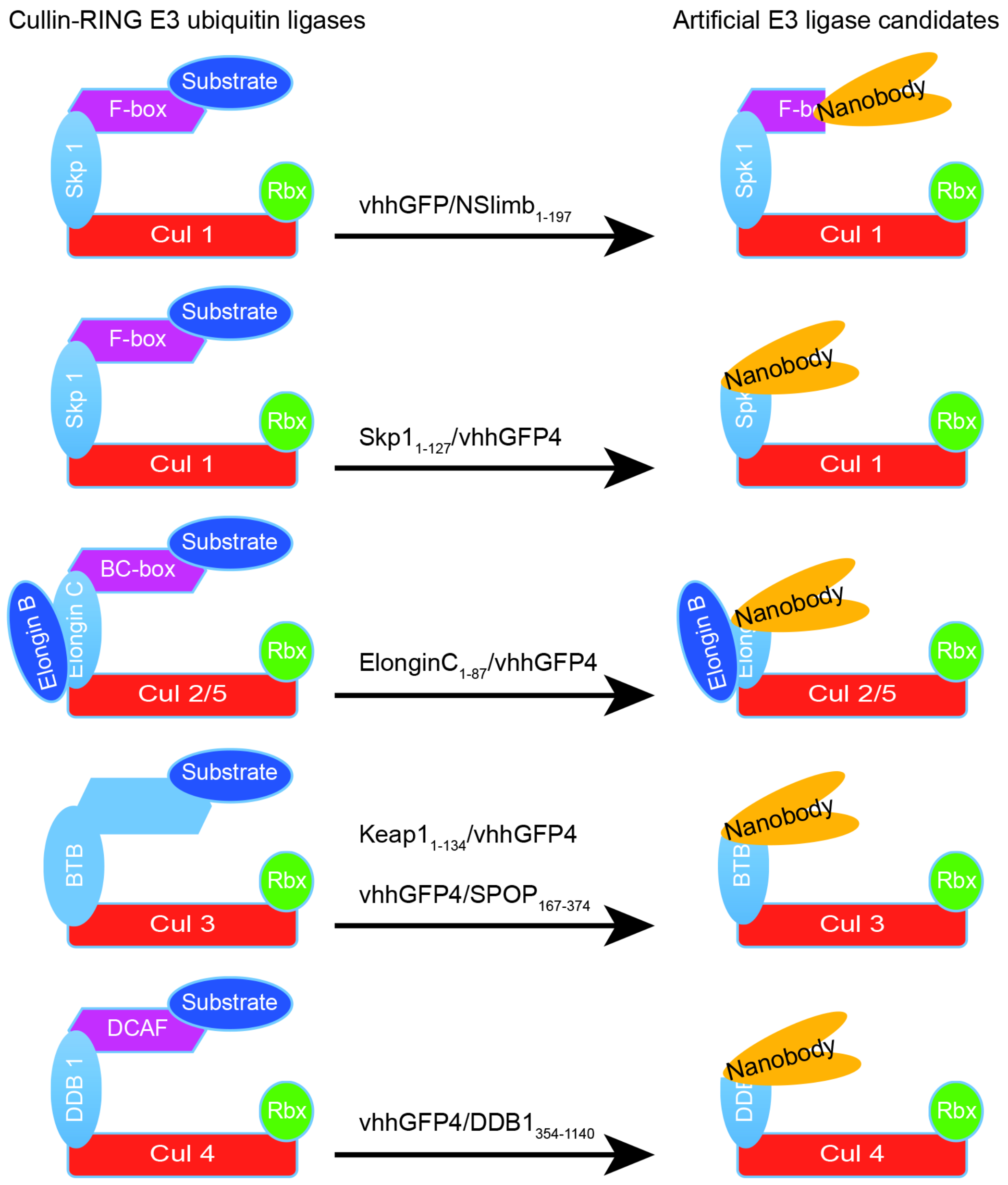

Supplementary Figure 1.
Design of synthetic E3 ubiquitin ligase candidates. In the deGradFP protocol, anti-GFP nanobody (vhhGFP4) was fused to NSlimb, an F-box protein, of the CRL1 E3 ligase complex. We generated synthetic E3 ligase candidates by fusing anti-GFP nanobody to adaptor proteins: Skp 1 for CRL1; Elongin C for CRL2/5; Keap1 and SPOP for CRL3; and DDB1 for CRL4. In each ligase candidate, the domain necessary for the interaction with substrate binding protein was replaced with the nanobody.

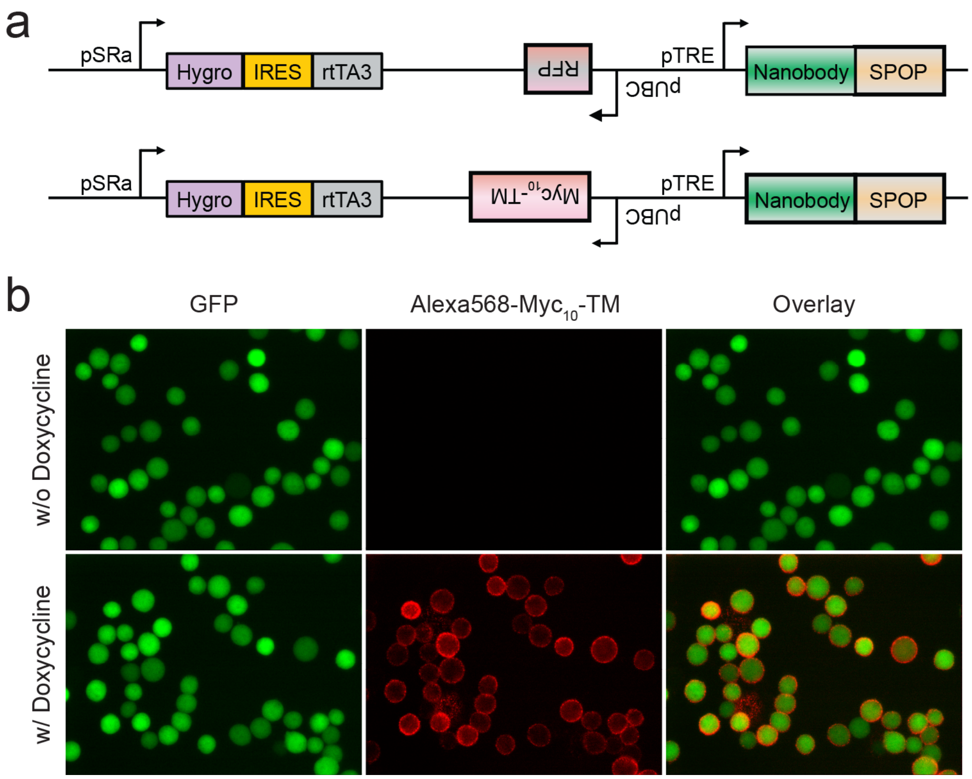

Supplementary Figure 2.
Bi-directional doxycycline-inducible promoters allow simultaneous expression of synthetic E3 ligases and Myc_{10}-TM (a) Each vector contains a bi-directional tetracycline response element (TRE) promoter, tetracycline repressor A3 (rtTA3), and TagRFP or Myc_{10}-TM tracer (10 tandem repeats of Myc epitope-transmembrane domain). **(b)** Following doxycycline treatment, Myc_{10}-TM was detected on the cell membrane by fluorescencent microscopy after immunostaining with anti-Myc primary antibody (9E10) and Alexa568-conjugated anti-mouse secondary antibody.

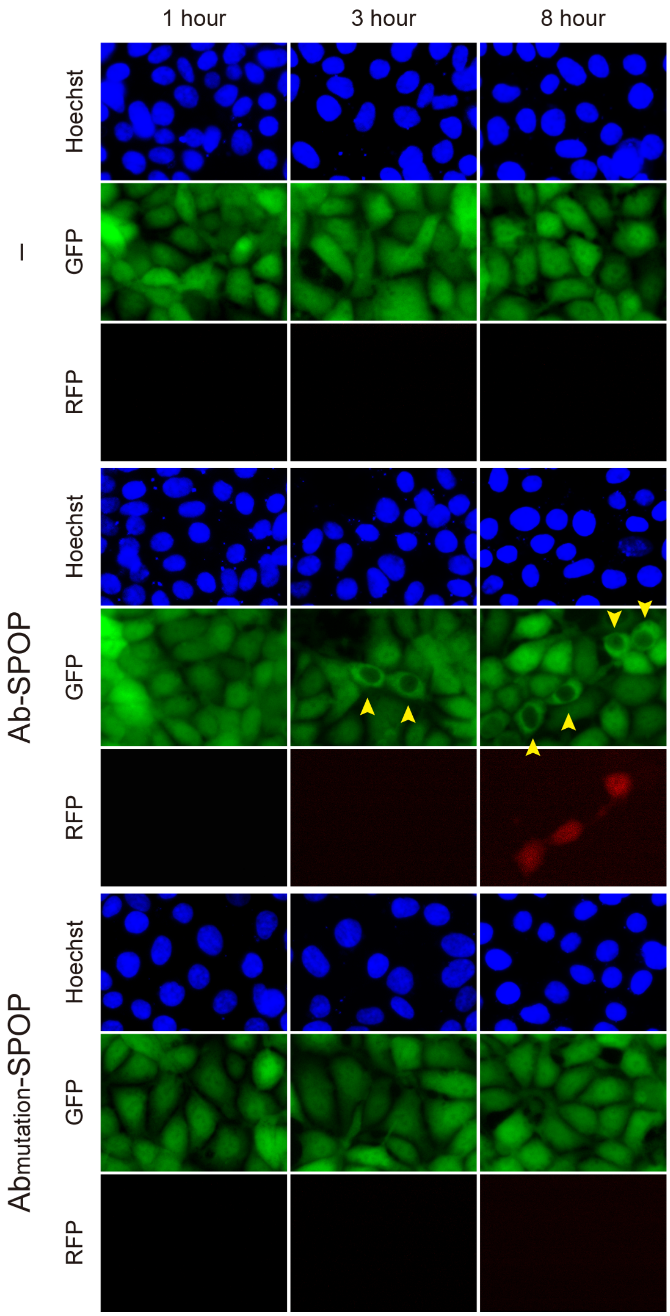

Supplementary Figure 3.

Depletion of GFP in nucleus, but not in cytoplasm, by Ab-SPOP. 293TetOn cells expressing GFP were transfected with vectors expressing Ab-SPOP$_{\Delta NLS}$ or Ab$_{mutation}$-SPOP$_{\Delta NLS}$ a bi-directional TRE promoter. '–' indicates untransfected cells . Expression of TagRFP and depletion of GFP were measured after adding doxycycline (1 μg/ml) to media. TagRFP was first detected about 8 hours after induction of TRE promoters by doxycycline. Yellow arrowhead indicates cells in which GFP is depleted in the nucleus, but not in cytoplasm. Nuclear depletion of GFP started 3 hours after activation of TRE promoters and was only observed in cells transfected with Ab-SPOP.

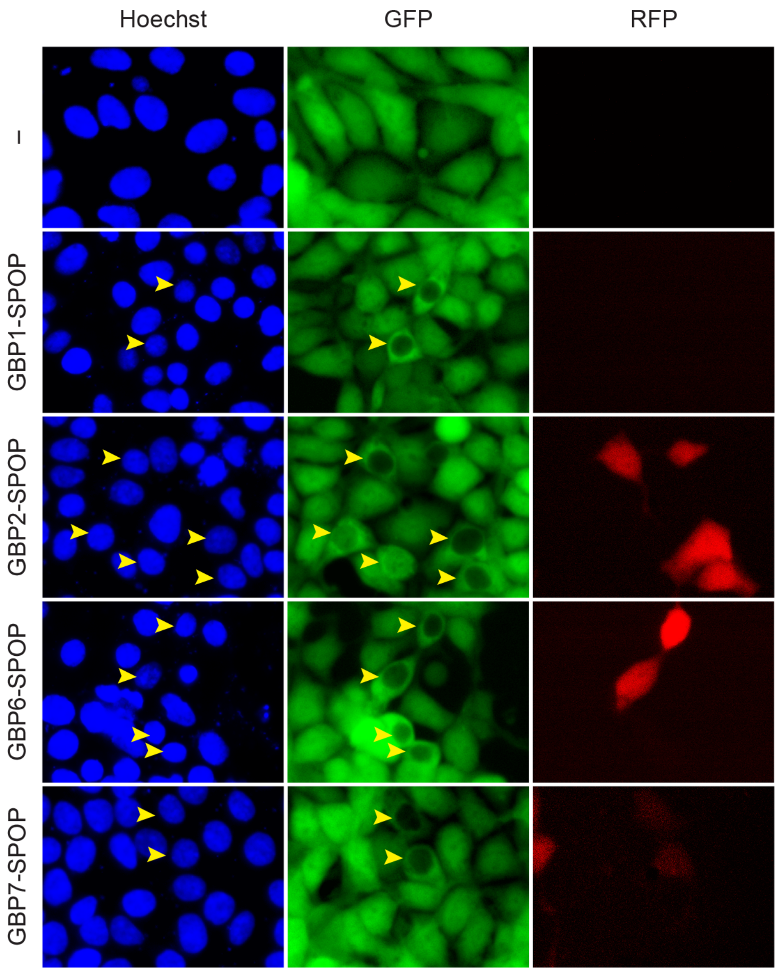

Supplementary Figure 4.

Selective depletion of nuclear GFP by Ab-SPOP E3 ligases engineered with other nanobodies against GFP. 293TetOn cells expressing GFP were transfected with vectors expressing four different Ab-SPOP$_{\Delta NLS}$ ligases (GBP1-SPOP, GBP2-SPOP, GBP6-SPOP, and GBP7-SPOP) from a bi-directional TRE promoter. Expression of TagRFP and depletion of GFP were measured 8 hours after adding doxycycline (1 μg/ml) to media. '–' indicates untransfected cells. Yellow arrowhead show cells where nuclear, not cytoplasmic GFP is depleted.

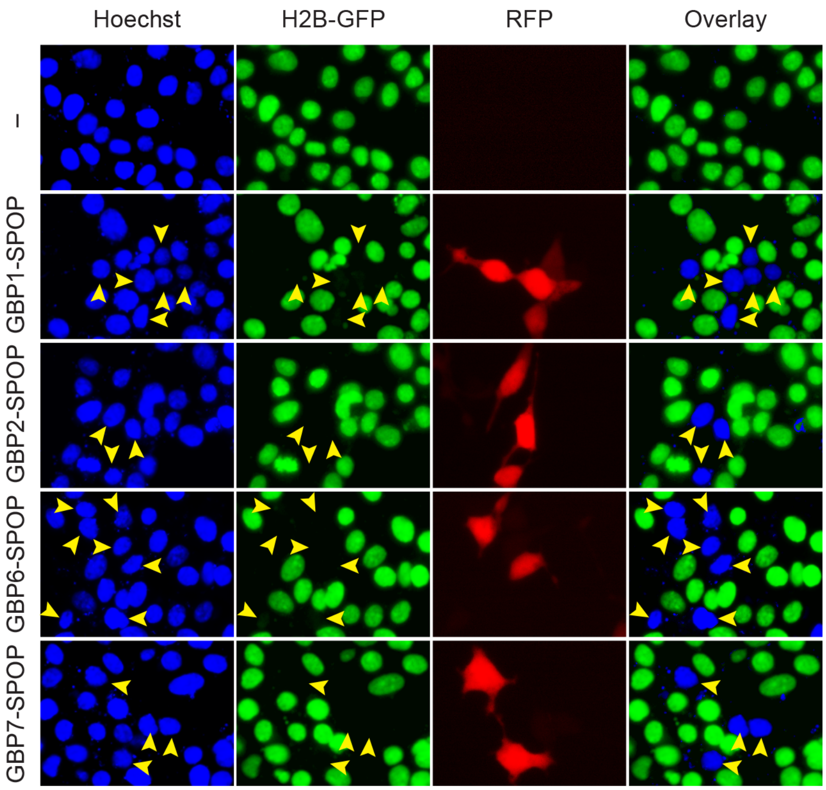

Supplementary Figure 5.
Depletion of H2B-GFP by Ab-SPOP E3 ligases engineered with alternative GFP nanobodies. 293TetOn cells expressing H2B-GFP were transfected with vectors expressing four different Ab-SPOP ligases (GBP1-SPOP, GBP2-SPOP, GBP6-SPOP, and GBP7-SPOP) from the bi-directional TRE promoter. Expression of TagRFP and depletion of H2B-GFP were measured 8 hours after adding doxycycline (1 μg/ml) to media. '–' indicates untransfected cells. Blue signal in the merged Hoechst/H2B-GFP panel indicates cells with depleted nuclear H2B-GFP. All cells expressing TagRFP had depleted H2B-GFP, however some cells with depleted H2B-GFP did not express TagRFP, consistent with the observation that nuclear depletion begins 3 hours after activation of the TRE promoter (**Supplementary Figs. 3** and **6**).

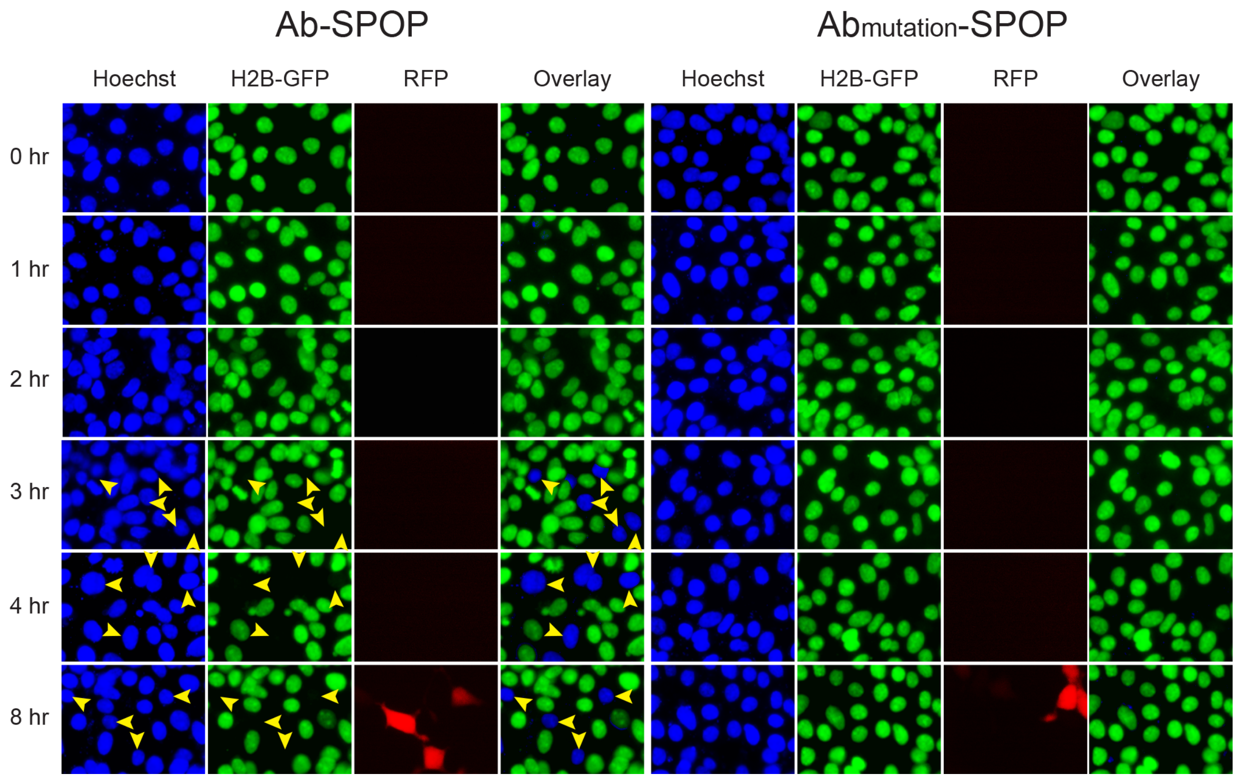

Supplementary Figure 6.

Time-course of H2B-GFP depletion by Ab-SPOP. 293TetOn cells expressing H2B-GFP were transfected with vectors expressing Ab-SPOP ligase (vhhGFP4-SPOP) or $Ab_{mutation}$-SPOP from bi-directional TRE promoters. Expression of TagRFP and depletion of nuclear H2B-GFP were measured after adding doxycycline (1 μg/ml) to media. Yellow arrowheads show nuclei with depletion of H2B-GFP. TagRFP was first detected about 8 hours after doxycycline-mediated TRE promoter activation. Depletion of nuclear H2B-GFP started 3 hours after TRE promoter activation and was observed in cells transfected with Ab-SPOP but not with $Ab_{mutation}$-SPOP.

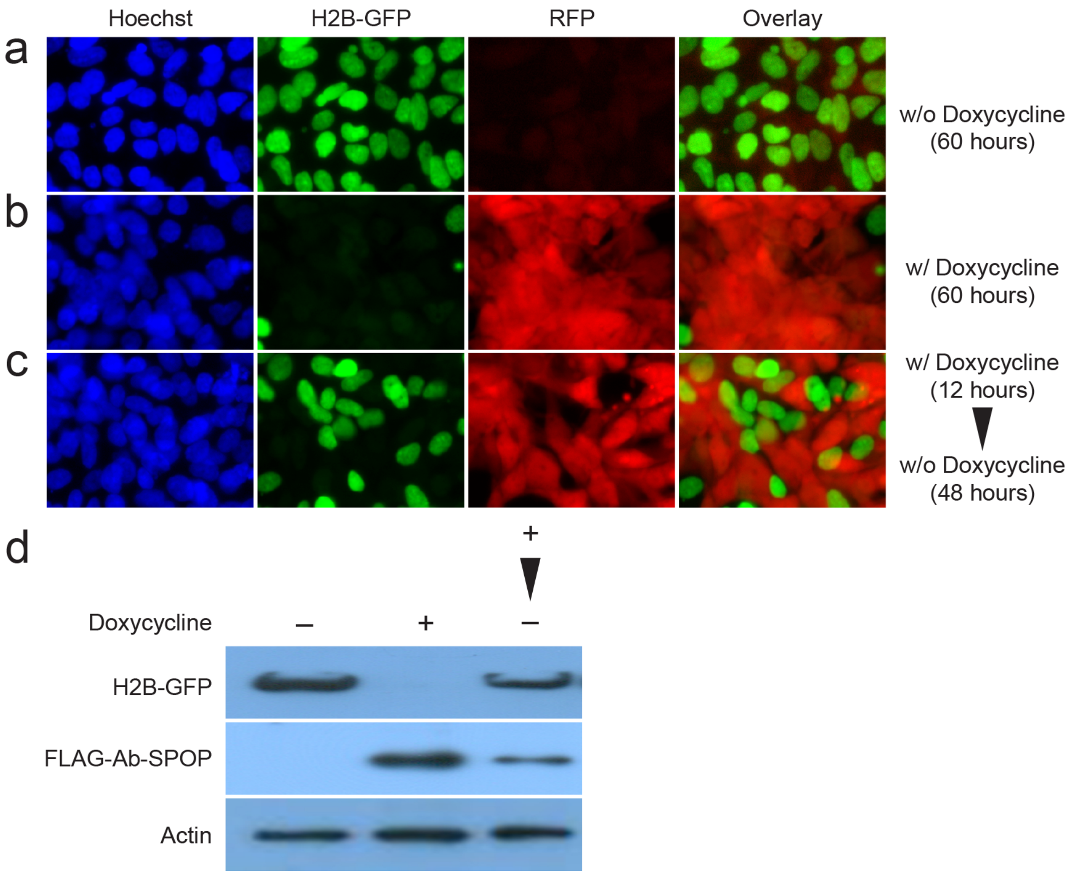

Supplementary Figure 7.

Reversibility of Ab-SPOP. 293TetOn cells expressing H2B-GFP from a CMV promoter and Ab-SPOP/TagRFP from a bi-directional TRE promoter recover H2B-GFP signal after doxycycline withdrawal. **(a)** Cells grown for 60 hours in medium without doxycycline. **(b)** Cells grown for 60 hours in medium containing doxycycline (1 μg/ml). Medium was changed every 24 hours. **(c)** Cells treated with doxycycline (1 μg/ml) for 12 hours, and then changed to fresh medium containing no doxycycline. Medium was changed every 24 hours. After 48 hours in medium without doxycycline, the H2B-GFP signal was partially recovered. **(d)** Protein extracts from the cells treated with doxycycline (–: w/o doxycycline for 60 hours, +: w/ doxycycline for 60 hours, '+ ⇒ –': w/ doxycycline for 12 hours and then with fresh medium for 48 hours) were analyzed by immunoblotting with anti-GFP, anti-FLAG, and anti-Actin antibodies.
